# A new strategy for constructing alternative consumer confidence indexes to explain household consumption: A fuzzy DEMATEL approach

**DOI:** 10.1016/j.heliyon.2024.e41447

**Published:** 2024-12-27

**Authors:** Özge Var, Alptekin Durmuşoğlu, Türkay Dereli

**Affiliations:** a Department of Industrial Engineering, Gaziantep University, Gaziantep, Turkey; b Department of Industrial Engineering, Samsun University, Samsun, Turkey; c Office of President, Hasan Kalyoncu University, Gaziantep, Turkey

**Keywords:** Consumer confidence index, Consumer surveys, Fuzzy DEMATEL, Household consumption, Lasso regression

## Abstract

**Background:**

Consumer Confidence Index (CCI) is a measure obtained from consumer surveys (CS) that gauges assessments and expectations of the economic environment. Common practice uses 4 of the 12 questions in CCI calculation. However, efforts to find best set of questions continue, such as the European Commission swapping two questions in 2019. Literature studies employ different combinations of questions; however all-alternative combinations take too much time and computational power. The questions also exhibit cause-and-effect relationships as household consumption predictors and are not statistically independent of one another.

**Objective:**

We suggest classifying the CS questions as "Causes" and "Effects." It makes sense that inquiries in the cause group should provide a better explanation of household consumption. If this theory turns out to be correct, a smaller solution space will be able to be used to find the ideal substitute CCI.

**Method:**

A fuzzy DEMATEL (Decision-Making Trial and Evaluation Laboratory), a reliable method to present causal relationships, is used to classification. The prediction power of cause group (in terms of explaining household expenditures) is measured with the Lasso regression (Least Absolute Shrinkage and Selection Operator), which provides more interpretable regression models. This approach was applied to European Union dataset from 2007Q3 to 2021Q2.

**Results:**

The cause group included four CS questions and explained the 75% variability of the consumption expenditures. It is performed comparably to earlier studies that took into account all possible question combinations. The Türkiye case, covering data from 2007 to 2021, supported the finding of EU case, explaining 84% variation in consumption expenditures.

**Conclusion:**

These encouraging results suggest that comparable prediction power can be attained with a significant reduction in effort (in comparison to all brute force). Therefore, this approach would provide shortcut for constructing alternative CCIs to the authorities.

## 1 Introduction

Economic decisions pertaining to household consumption are crucial. Purchasing intentions are quite important for forecasting household consumption, which seen as a proxy of economic activity. Over time, consumption behaviour is a key factor in determining economic growth, but in the short term, changes in consumption cause business cycles [1]. Decision-makers in the public and private sectors must constantly monitor the state of the economy [2] and it is essential for economic agents to accurately and promptly monitor economic conditions [3]. Thereby, recessions [4] and economic recoveries [5] can be predicted. The Consumer Confidence Index (CCI) is one of the best indicators to track the changes in the expectations and assessments about general economy and household economy of consumers. It is defined as the degree of optimism on the current state of the economy [6].

The European Commission (EC) oversees the coordination of Consumer Surveys (CS) carried out by authorized entities in all EU members and candidates [7]. The CS covers statements about future expectations, asked periodically (monthly and quarterly) on a) general economic situation, b) financial situation of household, c) unemployment levels, d) personal savings, and e) intended purchases. Every statement in the survey has response options that range from "very positive" to "very negative." Balances (difference between positive and negative answering options measured as percentage points of total answers) are computed for each question in order to obtain an overall score (aggregation of answers from multiple individuals) [8].

Authorities use the arithmetic mean of the four question balances—which they have determined to be the most useful set of indicators for illuminating household consumption—to calculate the CCI. These questions are namely the ones about: a) expected financial situation of household, b) expected general economic situation, c) unemployment expectations (with inverted sign), and d) expected savings all over the next 12 months.

Four out of the twelve questions found in CSs are typically used to measure consumer confidence accurately. However, the optimal set of questions to successfully arrive at the intended outcome is still up for debate. To illustrate their continuous search for the ideal set, the EC changed the index calculation as recently as 2019 by switching two questions. The revised index covers the questions on “past financial situation of household”, “expected financial situation of households”, “expected general economic situation”, and “intentions to make major purchases over the next 12 months”.

A few studies in the literature have also used various combinations of those questions and assessed how well they predict household consumption. Using various combinations of CS questions, while the 2047 alternative CCIs were constructed and analyzed by Jonsson and Linden [9], more than 86 million alternative confidence indicators were constructed in the study of Lolic et al. [10]. It is clear from previous studies that considering all alternative combinations of questions can be a time-consuming and computationally demanding task. Moreover, the importance of choosing the right set of questions for measuring consumer confidence cannot be underestimated. By choosing the right set of questions that accurately measure consumer confidence, decision makers can gain a better understanding of the economy and make more informed decisions.

**Fig. 1:**
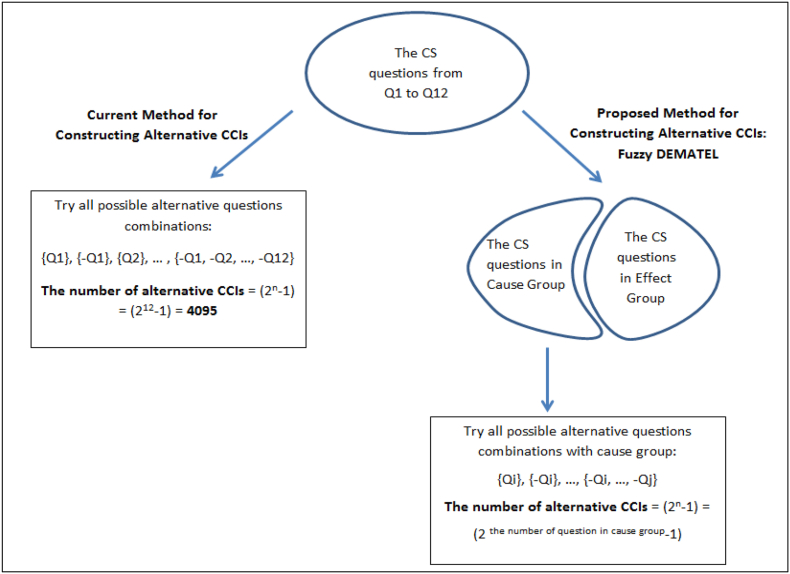
The comparison of traditional methods and proposed method to constructing alternative CCIs

Previous studies have looked at the optimal set of inquiries to represent household consumption. Nevertheless, there is no established process for choosing which CCI questions to include in order forecasting household consumption. Conventional methods emphasized trying nearly every combination from every question. Because each new CCI value corresponds to a different set of more than 4000 alternative questions, researchers were forced to analyze them (Fig. 1). This gap necessitates the re-evaluation of the constructing alternative confidence index process and the proposing the shortcut to existing time consuming brute forces.

The other key gap in the literature is that previous studies failed to notice both the direct relationship between the household consumption and the CS questions and the relationships among CS questions in constructing alternative confidence indexes. The CS questions exhibit a high degree of multi-collinearity and the predictive performance of the index is not repeatable in cross-sectional studies [11], even though they are not fully independent and have a cause-and-effect relationship. In addition to this, the household consumption and the questions that express intentions or sentiments about statements are related to cause and effect. Sorting the questions into cause and effect categories allows us to determine “which is the cause” and “which is the result.” However, it is important to note that the data gathered from CSs may have a limited sampling frequency (usually monthly or quarterly) along with delays in publication due to the time taken for collecting and analysing the responses [12]. Changes in individual expectations follow from changes in intentions, or in our case, expectations, which can be linked to changes in household consumption.

We have limited scope for this work because we are interested in the prediction power of a specific set of questions, not the effects of changes in household consumption. Effects don't give a coherent enough explanation to identify the best set that explains the change in it. Thus, the number of alternative survey questions that need to be examined will be greatly decreased if the cause-and-effect relationships between the questions are made clear (Fig. 1). With these purposes in mind, the research questions which are focused on this study are:

RQ1. What are the cause and effect relationship between CS questions and household consumption expenditures?
RQ2. What is the performance of the “causes” which put forward with the new approach for constructing alternative confidence indexes, in terms of explaining household consumption expenditures?

The “Fuzzy Decision Making Trial and Evaluation Laboratory (Fuzzy DEMATEL)” method is used for investigating the cause-and-effect relationships between CS questions in our analysis. By examining the logical connections between the system's components, this method integrates ideas from matrix theory and graph theory to create a direct influence matrix [13]. It has been a reliable and widely used method [14,15] to assess the cause-and-effect relationships [16]. Fuzzy is preferred because it can be used to quantify the degree of ambiguity in concepts related to subjective human judgment [17].

We study on European Union data set which covers the household consumption expenditures and the balances of CS questions from 2007Q3 to 2021Q2. The least absolute shrinkage and selection operator (Lasso) regression method, that is one of the widely used methods for variable selection to obtain more interpretable regression models [18], is used to evaluate the household consumption prediction performance of the questions in cause group. For comparing the prediction performance of the cause group, the lasso regression process is repeated with the questions in effect group, the set of questions used for calculating European CCI, the set of questions proposed in the study of Lolic et al. [10], and the set of questions proposed in the study of Jonsson and Linden [9] for EU. The robustness and reliability of this proposed approach was supported by the second data set which includes the household consumption expenditures and the balances of CS questions from 2007 to 2021 of Türkiye. For Türkiye case, the household consumption expenditures prediction performance of cause group compare with the effect group and official CCI.

We make three types of contributions to the literature. First, to our best knowledge, this paper is the first attempt to decompose CS questions as “Causes” and “Effects”. By filling this gap, our study makes a substantial progress in the removal of some question combinations when constructing alternative CCIs. Second, this paper is one of the few studies that focus on the direct relationships between household consumption expenditure and CS questions. Thus, questions in the cause group can be considered in constructing alternative CCIs without compromising the household consumption prediction performance. Thirdly, the fuzzy DEMATEL approach, which is infrequently documented for data pertaining to the economy, is employed in this study. This methodology affords the ability to discern the most salient criteria within the system as well as comprehend the causal relationships between the criteria.

The remainder of the study is organized as follows. The following section introduces the calculation of CCI and its literature background. The fuzzy DEMATEL method is explained in Section 3. While the results of the EU case are provided Section 4, the findings of the Türkiye case presented in Section 5. In Section 6, summary of the results, limitations, and opportunities for future studies are presented.

## 2 Calculation of consumer confidence index and its historical evolution

The calculation of CCI and literature on its household consumption prediction power are given in this section.

### 2.1 Calculation of consumer confidence index

The CCI is based on the Joint Harmonized EU Programme of Business and Consumer Surveys. The authorities in each EU member and candidate states have published the CCI in monthly frequency. The EC also produces aggregate confidence index results for the EU on the basis of the results received from the member states. About 32,000 consumers are surveyed every month across the EU. The sample size varies across countries according to the heterogeneity of their economies. The survey samples are supposed to register all the units of the whole population. The EU member countries use the stratified random sampling methodology to make the sampling process more efficient. For CS, the respondents are stratified according to sex, age, education, income and region of residence [19].

The monthly questions can be listed at Table 1. The four of the questions out of twelve questions, which are Q2, Q4, Q7, and Q11, are used for calculating European CCI. Since aforementioned questions lost their attractiveness for explaining the changes in household consumption, EC revised the set of questions included in CCI calculation. The revised CCI covers Q1, Q2, Q4, and Q9.

**Table 1:** Questions of consumer surveys

Q1) Financial situation of household at present compared to the last 12 months
Q2) Financial situation expectation of household over next 12 months
Q3) General economic situation at present compared to the past 12 months
Q4) General economic situation expectation over next 12 months
Q5) Assessment on consumer prices change rate over last 12 months
Q6) Expectation for consumer prices change rate over next 12 months compared to the past 12 months
Q7) Number of people unemployed expectation over next 12 months
Q8) Buying time condition of durable goods at present
Q9) Assessment on spending money on durable goods over next 12 months compared to the past 12 months
Q10) Saving time condition at present
Q11) The probability of saving over next 12 months
Q12) Statement on current financial situation of household

Questions in the survey have six answer options: situation is/will get ‘a lot better: very positive (PP)’, ‘better: positive (P)’, ‘the same: neutral (E)’, ‘worse: negative (M)’, ‘a lot worse: very negative (MM)’, and ‘do not know (N)’. The balances are calculated as the difference between percentages of positive responses and negative responses [2]. The ‘neutral responses’ and ‘do not know responses’ are omitted in the index calculation.

The balances are calculated by using Equation (1). B denotes “balance” which is calculated for each of predetermined set of questions (Question 1-2-4-9 in the last revised version). Very positive (PP) and very negative (MM) has one as a coefficient while positive (P) and negative (M) has ½ as coefficient (considering their relatively lower importance). The average of question balances (BQ_i_ shows the calculated the balance score of question i), gives the CCI score (Equation (2)).

(1) B=[PP+½P]–[½M+MM]

(2) $$\text{CCI}=\left[\left\{{\text{BQ}}_{1}+{\text{BQ}}_{2}+{\text{BQ}}_{4}+{\text{BQ}}_{9}\right\}/4\right]+100$$

To avoid having a negative score in the results, “100” is added to the average balance of four questions. Respectively, this means a value above 100 represents optimistic consumer confidence while a value below 100 represents pessimistic consumer confidence [20].

### 2.2 Literature on the prediction power of CCIs

Measuring CCI accurately has an important role on tracking wellness of the economy. Governments, manufacturers, retailers, and banks monitor changes in the CCI for decision-making processes about future household consumptions. Therefore, it is commonly utilized as an independent variable for explaining consumption growth (changes on GDP) in the literature [[21], [22], [23]].

There are several studies in the literature attempting to explain household consumptions with confidence indexes for different countries. The results of these empirical studies can vary attributed to the use of different data sources, different data collection frequencies, and timing of sampling. However, most of the studies confirm that CCI is a good indicator of household consumptions. In the study of Kwan and Cotsomitis [24], the confidence index is found as a useful tool for forecasting US household spending, especially during the periods of economic fluctuation. Cho [25] presented the same conclusion for Korea. The forecasting performance of the lagged values of CCI and the classical macroeconomic variables of consumption growth are compared in the study of Cho [25]. The confidence index explains variation in consumption growth 3.6% higher than the conventional macroeconomic variables. Bruno [26] questioned the household consumption forecasting ability of confidence index in Italy. While the confidence index is found significant for predicting durables and non-durables consumptions, it is not useful for forecasting semi-durable consumption.

The listed examples from literature have showed that the primary goal of the indicator is tracking macroeconomic data, especially consumption. Therefore, the EC reviews the performance of CCI on a regular basis [10]. There can be additional space for constructing alternative CCIs that are capable of predicting household consumption much more accurately.

Jonsson and Linden [9] analyzed the 2047 different alternative CCIs by considering different combination of CS questions before the revision of EC. They focused on finding alternative confidence indicator, which has highest correlation with the private consumption rate in EU, Euro Area (EA) and 25 EU countries. This study concluded that the best performing confidence indicators include the Q3, Q8, and Q9. The authors also hypothesized that a confidence indicator based on the questions related to household economy (micro-oriented) is more informative than the questions related to general economy (macro-oriented). However, the alternative confidence indicator includes the questions related to both macro-oriented and micro-oriented.

Since the CCI showed poor tracking performance in private consumption in some EU member states in a technical report of EC [19], the methodological improvement was made on the current CCI. The five alternative confidence indicators are compared before publishing the revised version of CCI. The correlation analysis, ability to track directional change, two in-sample models, an out of sample forecasting exercise and a volatility analysis are used for comparing the performances of alternative confidence indicators. The revised CCI is constructed with the questions Q1, Q2, Q4, and Q9 producing the best performance.

Claveria et al. [27,28] conducted two studies with using genetic programming to generate country specific business and consumer confidence index. The algorithm allows both an automated variable selection among CS questions and non-linear relationships between selected variables. They focused on 13 European countries and the EA in their first study. The derived CCI expressions involved non-linear interactions between survey questions. While some surveys questions appear in expression with ratios, some of them occasionally showed up for the same country both with and without lags. However, the proposed CCIs showed lower forecast errors in GDP growth. The analysis was repeated with the 19 European countries for different sample period on their second study in 2022. The results were similar with the first study. While the evolved expression of country specific CCIs are complicated, they showed good performance in tracking economic growth.

Lolic et al. [10] presented a study about the recent revision of the EU. They proposed the alternative best performing confidence index to explain consumption variable. In the analysis, they focused on the performances of alternative confidence indexes to track the changes in consumption direction. More than 86 million alternative confidence indicators were constructed with the different combination of CS questions. They found that the best-performing confidence index covers the questions: Q1, Q3, Q4, Q6, Q8, Q9, Q10, Q11, and Q12.

## 3 Methodology: Fuzzy DEMATEL

DEMATEL is a technique, which convert the interrelations between criteria into an intelligible structural model of the system. The graph theory and matrix tool are applied to examine criteria in complicated and structural systems [29]. The objectives of the method are determining the total causal relationships and categorizing the criterion as a cause or an effect [30]. It allows a better understanding of the cause role or effect role of the criterion in the system. The most important criteria in the system can also be detected with this method. It has been widely applied to identify the cause-and-effect factors of several systems such as customer buying decisions [31], portfolio of investment projects [32], and financial performance of industry [33].

Ullah et al. [34] summarized the advantages of DEMATEL in comparison to other multi criteria decision making (MCDM) models and statistical techniques. Unlike the factor analysis approach, DEMATEL does not assign equal weights to each category of criteria. Thus, it ensures that important criteria affecting system performance are revealed and these important criteria are focused on. DEMATEL does not require the assignment of equal and opposite weights in pairwise comparisons between criteria, as Analytic Hierarchy Process (AHP) and Analytical Network Process (ANP). The impact of criterion B on criterion A may differ from the impact of criterion A on criterion B in this manner. It appears to offer the advantages of requiring a limited number of data and considering all possible relationships among the criteria, as opposed to the regression method and structural equation modeling (SEM) [35].

In the DEMATEL, the system has a set of criteria. The initial causal relationships between these criteria are determined through expert opinions. Therefore, DEMATEL relies on the subjective opinions of experts [36]. The relationship between criteria is defined with five linguistic terms, which are “no influence”, “very low influence”, “low influence”, “high influence” and “very high influence” by the experts. These linguistics terms are denoted 0, 1, 2, 3, and 4, respectively in conventional DEMATEL technique.

Since CSs are replied by the human evaluators, inherently there is vagueness in their responses. To tackle the ambiguities involved in the process of human thinking and expression, the linguistic terms can be more effective in estimation [37]. Using linguistics terms such as “very high influence” etc. instead of using crisp numbers 0 to 4 is more convenient for experts when providing the paired comparison between criteria [38,39]. However, the conventional DEMATEL is not able to handle this vagueness and uncertainty in the responses of experts. To overcome this limitation, the fuzzy set theory has been incorporated with DEMATEL. The fuzzy DEMATEL method is applied with using fuzzy numbers to define the initial causal relationships between the criteria [40]. It can be utilized to measure the mutual effects of factors on one another [41]. With this hybrid method, the relationships among elements (also known as attributes/criteria) of mutual influence are denoted by the triangular fuzzy numbers (TFN). Each linguistics terms correspond to fuzzy numbers which have gradual memberships to sets of l(ow), m(edium), and h(igh) (Table 2). At the end of the analysis, the fuzzy numbers converted into crisp values (called as defuzzification) [14].

**Table 2:** Linguistic terms and corresponding TFN [42]

Linguistic terms	Triangular Fuzzy Numbers (l, m, h)
No influence	(0, 0, 0.25)
Very low influence	(0, 0.25, 0.50)
Low influence	(0.25, 0.50, 0.75)
High influence	(0.50, 0.75, 1.00)
Very high influence	(0.75, 1.00, 1.00)

**Fig. 2:**
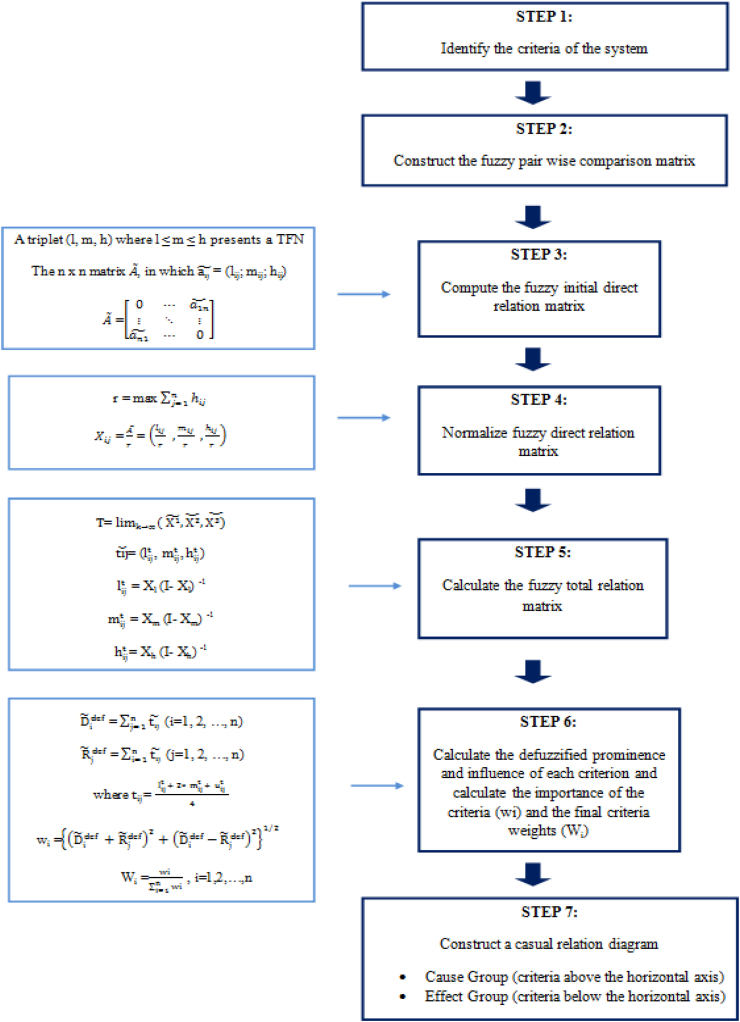
Flowchart of the fuzzy DEMATEL method steps.

The computational steps of the fuzzy DEMATEL method are explained in details as follows [[42], [43], [44]] and demonstrated by Fig. 2.

a) The criteria affecting the system are defined in Step 1. The expert opinion or literature review can be used in this step.
b) The fuzzy pairwise comparison matrix is constructed in Step 2. The criteria in Step 1 are presented to the experts. The experts assign degree of influence for each pair of criteria. The degrees of influences are represented with using five linguistic terms in the fuzzy scale.
c) Fuzzy initial direct relation matrix is prepared in Step 3. Each linguistic term corresponds to the positive TFN (Table 2).
d) Step 4 is the calculation of normalized fuzzy direct relation matrix.
e) Fuzzy total relation matrix is calculated in Step 5 where “I” represents identity matrix.
f) Prominence and influence of each criterion, which are denoted by the fuzzy numbers $\tilde{\left(\text{Di}\right)\;}$ and $\tilde{\left(\text{Rj}\right)}$, are presented in Step 6 respectively. They correspond to the sum of rows and sum of columns of the sub matrices $l_{ij}^{t}$ , $m_{ij}^{t}$ , and $h_{ij}^{t}$. In order to get the importance of criteria and the final criteria weights, defuzzification is required. Defuzzified $\tilde{\text{Di}}$ and $\tilde{\text{Rj}}$ are denoted by ${\tilde{D}}_{i}^{\mathrm{def}}$ and ${\tilde{\;R}}_{j}^{\mathrm{def}}$, respectively. The importance of the criteria (wi) and the final criteria weights are calculated [42].
g) In Step 7, a casual relation diagram is constructed with $\left({\tilde{D}}_{i}^{\mathrm{def}}+{\tilde{R}}_{j}^{\mathrm{def}}\right)$ being the horizontal axis, and $\left({\tilde{D}}_{i}^{\mathrm{def}}-{\tilde{R}}_{j}^{\mathrm{def}}\right)$ the vertical axis. When the criterion is located above the horizontal axis, it means that this criterion is a net causer. It is grouped in the cause cluster. In contrast, when the criterion is located below the horizontal axis, it means that this criterion is a net receiver.

## 4 Analysis and results of the European Union case

The results of the fuzzy DEMATEL, the lasso regression and performance analysis for EU case are given in this section.

### 4.1 The results of the fuzzy DEMATEL

The questions of the CS are accepted as the criteria of our model in explaining the household consumption. While the questions of CS are widely recognized and accepted factors of household consumption (literature section can be seen), we did not require further expert opinions. The total number of criteria in the analysis is equal to the total number of questions in the CS.

After defining the criteria of the system, the pairwise comparison matrix is prepared. In this step, correlation coefficients are used as the proxy of influence degree between criteria. Pearson's correlation coefficient is used to measure whether two data sets are on a line and is used to measure the linear relationship between the distance variables [43,45]. Even that is not the same with our work; Lolic et al. [10] previously used the correlation coefficient as evaluation/performance criteria for alternative confidence indexes (as an alternative of existing European CCI). Since the purpose of Lolic et al. [10] was to explain private consumption data (PCD), the alternative question combinations (alternative indexes) having the higher correlation with PCD was labeled as higher-ranking. If a CS question shows high relevance in constructing alternative confidence indicator, it demonstrates the strong rationale for inclusion in the new confidence indicator. While alternative set of questions are evaluated according to the correlations, this provides a logical support for using them as the pairwise comparison matrix.

The Pearson's correlation coefficient between the balances of the CS questions and household consumption from 2007Q3 to 2021Q2 are given in Table 3. Since we have the “household consumption” data for quarters in one hand, monthly balance of CS questions in other hand, to have compatible data, we converted the monthly balances into quarterly values (just simple average months).

**Table 3:** The correlation between the balances of survey questions and household consumption

Criteria Number	Question Number in the CS	Pearson’ s Correlation Coefficient (Sig. 2-tailed)
C1	Q1	0.80ʹʹ (0.000)
C2	Q2	0.69ʹʹ (0.000)
C3	Q3	0.50ʹʹ (0.000)
C4	Q4	0.36ʹʹ (0.006)
C5	Q5	−0.38ʹʹ (0.004)
C6	Q6	0.09 (0.525)
C7	Q7	−0.50ʹʹ (0.000)
C8	Q8	0.70ʹʹ (0.000)
C9	Q9	0.79ʹʹ (0.000)
C10	Q10	−0.52ʹʹ (0.000)
C11	Q11	0.85ʹʹ (0.000)
C12	Q12	0.78ʹʹ (0.000)
ʹʹ Correlation is significant at the 0.01 level (2-tailed).		

The significance and the direction of the correlation are not taken into account for our analysis. The relationship strength, which is defined as an absolute value of the correlation coefficient, is considered for constructing pairwise matrix. The construction of pairwise comparison matrix can be summarized as follows.

a) The difference between the relationship strength of the C1 (|0.80| = 0.80) and the relationship strength of the C4 (|0.36| = 0.36) is high. It is mean that C1 has very high influence on household consumption when compared to the influence of C4 on household consumption. In other words, the C4 has no influence on household consumption when comparing the influence of C1 on the household consumption.
b) The relationship strength of the C1 (|0.80| = 0.80) and C9 (|0.79| = 0.79) are equal. Therefore, the influence of C1 on household consumption and the influence of C9 on household consumption are the same and represented by low influence in the pairwise comparison matrix.
c) The relationship strength of the C1 (|0.80| = 0.80) is higher than the relationship strength of the C2 (|0.69| = 0.69). It means that, C1 has high influence on the household consumption when compared the influence of C2 on the household consumption. In other words, C2 has very low influence on the household consumption when compared the influence of C1 on the household consumption.

The fuzzified relationships between the criteria in the examples (a), (b), and (c) are illustrated in Table 4. The relationships are denoted linguistic terms (fuzzy pairwise comparison matrix) in the first part of Table 4. Fuzzy initial direct relation matrices, which include TFN corresponding to the linguistic terms, are given in the second part of Table 4.

**Table 4:** The fuzzy pair wise comparison matrixes and fuzzy initial direct relation matrixes of the examples (a), (b), and (c)

Part 1. The fuzzy pair wise comparison matrixes of the examples (a), (b), and (c)											
	**C1**	…	**C4**		**C1**	…	**C9**		**C1**	**C2**	…
**C1**	0	…	Very High Influence	**C1**	0	…	Low Influence	**C1**	0	High Influence	…
**…**	…	…	…	**…**	…	…	…	**C2**	Very Low Influence	0	…
**C4**	No Influence	…	0	**C9**	Low Influence	…	0	**…**	…	…	…
**(a)**					**(b)**				**(c)**		

**Part 2. The fuzzy initial direct relation matrixes of the examples (a), (b), and (c)**											

	**C1**	**…**	**C4**		**C1**	**…**	**C9**		**C1**	**C2**	**…**
**C1**	0	…	(0.75, 1.00, 1.00)	**C1**	0	…	(0.25, 0.50, 0.75)	**C1**	0	(0.50, 0.75, 1.00)	…
**…**	…	…	…	**…**	…	…	…	**C2**	(0, 0.25, 0.50)	0	…
**C4**	(0, 0, 0.25)	…	0	**C9**	(0.25, 0.50, 0.75)	…	0	**…**	…	…	…
**(a)**				**(b)**				**(c)**			

The pairwise comparisons of twelve criteria are completed according to this methodology and presented with using linguistic terms in Table 5 (in Appendix 1). The linguistic terms are converted the fuzzy numbers, given in Table 2, and fuzzy initial direct matrix is constructed. The normalized fuzzy direct relation matrix and fuzzy total relation matrix are calculated. Because of their big sizes, these matrices are not given in the study. The prominence and influence of each criterion, defuzzified Di and Rj values, the importance of the criteria and the normalized weight of each criterion can be found in Table 6 (in Appendix 2).

**Fig. 3:**
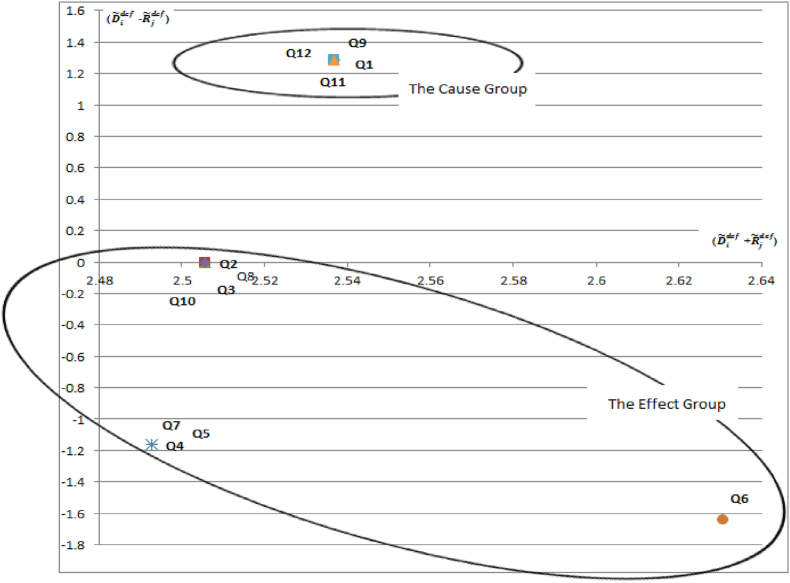
The causal relations diagram (${\tilde{D}}_{i}^{\mathrm{def}}$-${\tilde{R}}_{j}^{\mathrm{def}}$) versus (${\tilde{D}}_{i}^{\mathrm{def}}$ + ${\tilde{R}}_{j}^{\mathrm{def}}$)

The fuzzy DEMATEL results demonstrate that C2, C3, C4, C5, C6, C7, C8, and C10 are defined as receiver because of the their negative (${\tilde{D}}_{i}^{\mathrm{def}}$ - ${\tilde{R}}_{j}^{\mathrm{def}}$) values. It means that Q2, Q3, Q4, Q5, Q6, Q7, Q8, and Q10 are the factors, which get the most impact from other factors. The Q1, Q9, Q11, and Q12 have the most influence on other factors. For this reason, this set of questions is categorized a “cause” group. The causal relations diagram (Fig. 3) shows the cause-and-effect group. Since the Q1, Q9, Q11, and Q12 are categorized as “cause” group, this set of questions is candidates of being a part of alternative confidence indicators.

When the questions of the cause group are inspected, it is found that the three questions are related to the assessments about “financial situation” and “making major purchases”. Only, the Q11 is related to the expectations about the saving. This finding indicates that the questions measuring the “assessments” of consumers have a greater influence on household consumption when compared to the questions on “expectations” of consumers.

The all questions in cause group are related to household financial economy. It is indicated that the micro-oriented questions affect household consumption more than macro-oriented questions. This finding is in line with the hypothesis in the study of Jonsson and Linden [9]. They were unable to confirm this hypothesis. However, our findings confirm the hypothesis and show that survey respondents are more aware of their personal financial circumstances than the overall state of the economy.

The importance of the criteria can be prioritized as Q6 > Q1 = Q9 = Q11 = Q12 > Q4 = Q5 = Q7 > Q2 = Q3 = Q8 = Q10 based on the final criteria weights (Wi). The “Q6- Expectation for consumer prices change rate over next 12 months compared to the past 12 months” has highest final criteria weight. It means that it is the most important criterion in the system. Since Q2, Q3, Q8, and Q10 have the lowest final criteria weight, they are the least significant criteria in the system. The importance levels of the questions in cause group are equal according to our results. This finding coincides with the CCI calculation methodology of EC. The average of question balances gives the CCI score and not provided any weighting application in the calculation of index. The equal importance levels of the questions in the cause group also indicated that there is no requirement for weighting.

The similarities between the set of questions of European CCI, the question sets proposed in the literature and the cause group are summarized in Table 7. The two questions in the cause group, which are Q1 and Q9, are also in the question set of the European CCI. That is a kind verification of similar findings of two different approaches.

Although the process underlying Lolic et al. [10] and our investigation are distinct, the findings also point to certain parallels. The proposed set of questions for alternative confidence indicator in Lolic et al. [10] includes nine questions, which are Q1, Q3, Q4, Q6, Q8, Q9, Q10, Q11, and Q12. The four of them, which are Q1, Q9, Q11, and Q12, are also in the cause group in our analysis. When the cause group is compared with the question set proposed in Jonsson and Linden [9], Q9 is included in both question sets, only.

### 4.2 The results of the lasso regressions

By using the fuzzy DEMATEL, we categorize the 12 CS questions as “causes” or “effects”. Our claim also implies that the best descriptor of the household consumption should be the ones (questions) that are in “causes” category not the ones in “effects” category. Given that four questions in the cause group, we can construct 480 alternative confidence indexes. It also means that 480 possible regression models can be developed to calculate their household consumption prediction performances. If we want to compare the prediction performance of cause group with the effect group, all possible alternative confidence indexes are needed to construct with the effect group questions. It is clear from previous researches that considering all alternative combinations of questions can be time-consuming and computationally demanding task. For this reason, to find the household consumption prediction performance of cause group, we utilize the least absolute shrinkage and selection operator (Lasso) regression method.

The lasso regression is one of the widely used methods for variable selection to obtain more interpretable regression models [18]. This model chooses a subset of the available independent variables with setting some model coefficient to zero. Therefore, it decreases the dimension of the model [18] and solves the multi-collinearity problem, which frequently encountered in multiple linear regressions [46]. The estimator of the lasso regression is given in Equation (3).

(3) $${\hat{\beta }}_{lasso}=\arg_{\beta }\;\min\left\{\sum_{i=1}^{n}{\left(y_{i}-\beta_{0}-\sum_{j=1}^{p}x_{ij}\beta_{j}\right)}^{2}+\lambda \sum_{j=1}^{p}\left|\beta_{j}\right|\right\}$$

In Equation (3), n is the number of observations, y is the dependent variable, p is the number of variables, *β*_0_ and *β*=(*β*_1_,*β*_2_, …,*β*_*p*_) are the unknown parameters, *x*_*i*_=(*x*_*i*1_,*x*_*i*2_, …,*x*_*ip*_)^*T*^ are the independent variables and *λ* is the penalty term. The penalty term (*λ*) is the parameter that controls the amount of shrinkage. If the value of penalty term increases, the amount of shrinkage increases at the same rate [46]. The choice of the subset of the available independent variables depends on the penalty term. To find the optimum value for the penalty term which minimize the error of the fitted model, K-fold cross validation is widely used. The sampled units are divided into K equal sized subsamples at random using this procedure. While the Kth subsample is designated as the test set, the remaining K-1 subsets form the training set. Next, a grid for penalty term is defined. A model is fitted to each training set and applied to the test set for each of these values. This procedure is carried out K times with using a different Kth subset as the test set. Finally, the penalty parameter which minimizes the cross-validated error is selected [18].

The expression *l*_1_ = $\sum_{j=1}^{p}\left|\beta_{j}\right|$ is called the penalty function. The value of the penalty function affects the number of variables that will enter the regression model. If the value of *l*_1_ increases, the number of variables that enter the model increases [46].

In the lasso regression analysis, we used the household consumption expenditure as a dependent variable. The balances of questions in the cause group construct the set of independent variables. The results of the lasso regression model for EU case are given in Table 8. The part (a) proposed the lasso regression penalty parameter and the independent variables whose coefficients are set to zero. The part (b) shows the effect of reducing the number of independent variable to the fitting model. Since the lasso variable selection uses a different measure (AIC) to select preferred model, the coefficients of independent variables and R-squared in the part (b) show little differences from the part (a).

The whole data set is from 2007Q3 to 2021Q2 for EU case. Therefore, the number of observation is 56. The 10-fold cross validation is used to select lambda (penalty parameter). The all coefficients at the minimum value of lambda (57.64) are nonzero. However, when the lambda value is moved to 1.08e+05, which is the largest value of lambda that is within one standard deviation of the minimum, two of coefficients are nonzero. If the lambda moves to two standard deviation of the minimum, the independent variables with nonzero coefficient are not changed. The adjusted R-squared is 0.75 with two independent variables. The Q1 and Q11 account for 75% of the variance in the household consumption expenditures.

The household consumption prediction performance of effect group, the question set of European CCI, the questions set proposed in Lolic et al. [10] and Jonsson and Linden [9] are also given in part (a) of Table 9 (in Appendix 3). The effect group includes eight questions of CS according to fuzzy DEMATEL method. In lasso regression, only, the coefficient of the Q7 is set to zero. The remaining seven questions explain the 84% of variation of household consumption expenditures. The explanation performance of effect group is better than the cause group. However, the model constructed with cause group, provided this prediction performance with two independent variables while the model constructed with effect group, with seven independent variables. Since the model constructed with cause group requires a limited number data, it shows better performance than the model constructed with effect group.

When the set of questions, which are used in calculation of European CCI, is used lasso regression model, only Q1 enters the regression and explains the 64% variation of household consumption. When compared the model constructed with European CCI, the model constructed with cause group requires more input data. However, it shows better explanation performance than the European CCI.

When the performances of the two studies in the literature are reviewed, the question set proposed in Lolic et al. [10] has the best performance in prediction of household consumption expenditures. However, its data requirement is more than our proposed cause group model. Therefore, it can be concluded that, our proposed model shows better prediction performance with limited data. If we compare the performances of cause group and the question set proposed in Jonsson and Linden [9], the model constructed with the question set proposed in Jonsson and Linden [9] requires more input data than the model constructed with cause group. Its performance in explaining household consumption is also lower than cause group. It is summarized that, the explanation power of cause group is better than the question set of European CCI and the question set proposed in Jonsson and Linden [9]. The effect group and the question set proposed in Lolic et al. [10] show better explanation performance than cause group. However, the requirements of input variables for these models are higher than our proposed cause group.

### 4.3 The performance analysis of proposed model

We evaluate the performance of our model according to two performance indicators which are “the number of possible alternative CCIs” and “the explanation power of household consumption”. It is summarized in Table 10 as pairwise comparison with the studies in literature.

**Table 7:** Comparison of the question sets with cause group

	Q1	Q2	Q3	Q4	Q5	Q6	Q7	Q8	Q9	Q10	Q11	Q12
Cause Group	+								+		+	+
European CCI	+	+		+					+			
Lolic et al. [10]	+		+	+		+		+	+	+	+	+
Jonsson and Linden [9]			+					+	+

**Table 8:**
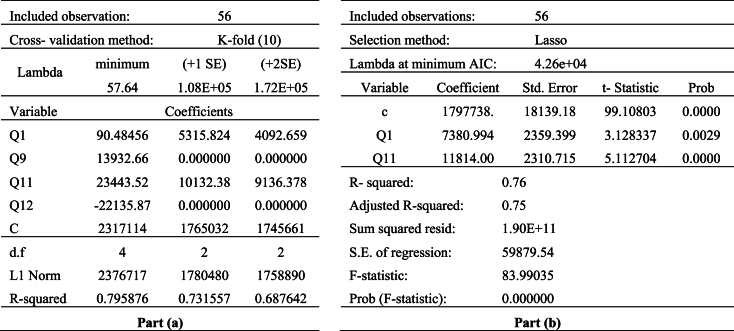
Results of lasso regression with cause group of EU case.

**Table 10:** The performance comparison of models in literature with cause group of EU case

Cause Group				
	The Number of Alternative CCIs	The % Change in The Number of Alternative CCIs	The Adjusted R^2 (Lasso Regression)	The % Change in Adjusted R^2 (Lasso Regression)
Effect Group	39,366	−98.78	0.84	−10.60
European CCI	480	0.00	0.64	18.25
Lolic et al. [10]	86,093,436	−100,000	0.88	−14.52
Jonsson and Linden [9]	2047	−76.55	0.66	13.75

When calculating the alternative CCIs, Lolic et al. [10] used Equation (4). The “n” is represent the number of questions in CS, the “3” represents the situations for questions “the included in the index with a positive sign” or “a negative sign” or “not included in the index”. The second multiplier, which is equal to “3”, represents the three ways of calculating quarterly values from monthly values. The “2” represents the situations “applied standardization” or “not applied standardization”. According to this equation 86,093,436 alternative CCIs can be constructed with 15 CS questions (12 monthly and 3 quarterly questions). We used this equation to find the number of alternative CCIs constructed with cause group and effect group. While the number of alternative CCIs constructed with the questions in cause group corresponds to 480, it corresponds to 39,366 alternative CCIs for the effect group.

(4) $$\text{The}\;\text{number}\;\text{of}\;\text{possible}\;\text{alternative}\;\text{CCIs}=\left(3^{n}‐1\right)*3*2$$

Jonssons and Linden [9] used Equation (5) to construct alternative CCIs. The “n” represents the number of questions. The “2” corresponds to the situations for questions “included in the index” or “not included in the index”. Therefore, they studied on 2047 alternative CCIs (11 monthly questions).

(5) $$\text{The}\;\text{number}\;\text{of}\;\text{possible}\;\text{alternative}\;\text{CCIs}=\left(2^{n}‐1\right)$$

Since our model limits the question set with four questions for EU case, it is provided a significant decreasing in the number of alternative CCIs. It means that our model will require least computational power and time to find the best set of question in explaining household consumption. Given that the performance of effect group, the cause group will provide 98.78% decreases in the number of alternative CCIs while provide 10.6% decreases in explaining household consumption. Our model shows better performances in both performance indicators when compared to proposed question set in Jonsson and Linden [9] and European CCI. The proposed set of Lolic et al. [10] shows better performance in household consumption explanation than our model. However, our model provides a significant decrease in the number of alternative CCIs. In other words, our model needs less computational power and time than Lolic et al. [10]. Therefore, the 14.52% decrease in household consumption explanation power is acceptable.

## 5 The validation of the proposed model: Türkiye case

After obtaining the fuzzy DEMATEL results of the EU case, we compared the household consumption expenditures explanation power of the cause group with the effect group, European CCI, and the other proposed questions sets in the literature in order to validate the rationality of our approach. Then, we define two performance indicators to verify the advantage of our method in constructing alternative confidence indicators. Finally, we repeated all technical steps of our approach for Türkiye data set in this section to show the reliability and the robustness of fuzzy DEMATEL method.

Türkiye is the one of the candidate states of EU. To harmonize with the EC Program, the Consumer Tendency Survey has carried out in cooperation with the Central Bank of the Republic of Turkey (CBRT) and Turkish Statistical Institute (TURKSTAT) from January 2004. It is conducted through computer-based; face-to-face method within the first two weeks of the each month. It is released on the third week of the each month. The sample size for Turkey is 4884 individuals, which cover people selected randomly by the data entry program [6].

Türkiye also tracks the revision on the CCI of EC. This revision is applied to the old series value of the CCI from January 2004 to September 2020. The difference between the new series values of the official CCI and the old series values of the official CCI is calculated 19.36% on average [47]. It indicates a considerable change into the series. Therefore, to consider studies related to confidence index for candidate countries is as important as to consider confidence index studies for EU.

### 5.1 The results of the fuzzy DEMATEL: Türkiye case

The questions of the CS are accepted as the criteria of the model for Türkiye case like the EU case. The pairwise comparison matrix is prepared according to the Pearson's correlation coefficients between criteria. The data span covers the balances of the CS questions and household consumption from 2007 to 2021. Since we have the “household consumption” data for annuals, the monthly balances are converted into annual values (just simple average months). The pairwise comparisons of twelve criteria are completed based on the correlation coefficients with using linguistic terms. These linguistic terms are converted the fuzzy numbers and fuzzy initial direct matrix is constructed. The normalized fuzzy direct relation matrix and fuzzy total relation matrix are calculated. Because of their big sizes, these matrices are not given in the study. The prominence and influence of each criterion, defuzzified Di and Rj values, the importance of the criteria and the normalized weight of each criterion can be found in Table 11 (in Appendix 4).

The fuzzy DEMATEL results demonstrate that C2, C4, C5, C6, and C8 have the most influence on other factors because of the their positive (${\tilde{D}}_{i}^{\mathrm{def}}$ - ${\tilde{R}}_{j}^{\mathrm{def}}$) values. It means that this set of questions is categorized a “cause” group. The two questions in the cause group, which are Q2 and Q4, are also in the question set of the official CCI. However, it is completely different from the proposed cause group for EU case in this study. This differentiation supports the idea of constructing country specific consumer indicators in Claveria et al. [27,28].

The Q2, Q4, and Q6 are related to the expectations about “financial situation” and “general economic situation” while the Q5 and Q8 are related to the assessments about “general economic situation”. This finding indicates that the questions measuring the “expectations” of consumers have more influence than “assessments” of consumers on household consumption for Türkiye. The four questions in cause group, which are Q4, Q5, Q6, and Q8 are related to general economy. It is indicated that the macro-oriented questions affect household consumption more than micro-oriented questions for Türkiye. The importance of the criteria can be prioritized as Q8 = Q5 > Q1 = Q3 = Q7 = Q9 = Q10 = Q11 = Q12 > Q2 = Q4 = Q6 based on the final criteria weights (Wi). The “Q5- Assessment on consumer prices change rate over last 12 months” and “Q8- Buying time condition of durable goods at present” are the most important criteria in the system according to their highest final criteria weight. Since Q2, Q4, and Q6 have the lowest final criteria weight, they are the least significant criteria in the system.

### 5.2 The results of the lasso regression: Türkiye case

In the lasso regression analysis, we used the household consumption expenditure as a dependent variable. The balances of questions in the cause group construct the set of independent variables for Türkiye. The results of the lasso regression model are given in Table 12. The whole data set is from 2007 to 2021, annually. Since the number of observation is low, the 5-fold cross validation is preferred to select penalty parameter.

**Table 12:**
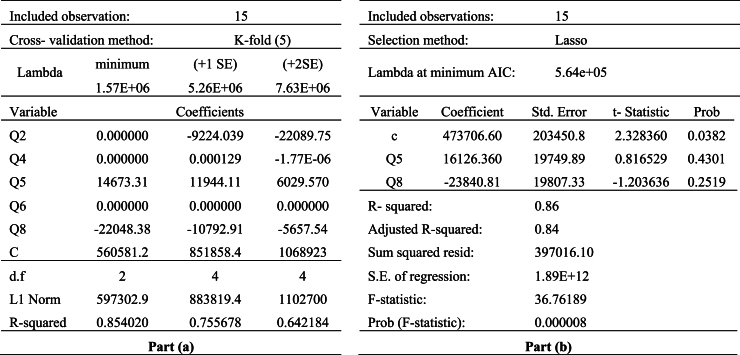
Results of lasso regression with cause group of Türkiye case.

The coefficients of Q5 and Q8 are nonzero at the minimum value of lambda (1.57e+06). When the lambda value is moved to one standard deviation of the minimum, L1 norm increases. Therefore, more coefficients enter the model. For the lambda value within two standard deviation of the minimum, while L1 norm increases, R-squared decreases. For this reason, the best lasso model for cause group of Türkiye case is the lasso model with minimum lambda. The adjusted R-squared is 0.84 for the model with two independent variables. The Q5 and Q8 account for 84% of the variance in the household consumption expenditures.

To our best knowledge, there is not any study related to construct alternative CCIs for Türkiye in literature. Therefore, for Türkiye case, the household consumption prediction performance of effect group and the question set of official CCI are given in part (b) Table 9 (in Appendix 3). The effect group includes seven questions of CS according to fuzzy DEMATEL method. In lasso regression, only, the coefficient of the Q3 and Q11 are set to nonzero. These two questions explain the 46% of variation in household consumption expenditures. The model constructed with cause group and the model constructed with effect group have the same data requirements. However, the explanation performance of effect group is lower than the cause group. Therefore, the model constructed with cause group shows better performance than the model constructed with effect group.

When the set of questions, which are used in calculation of official CCI, is used lasso regression model, only Q2 enters the regression and explains the 63% variation of household consumption. When compared the model constructed with official CCI, the model constructed with cause group requires more input data. However, it shows better explanation performance than official CCI.

### 5.3 The performance analysis of proposed model: Türkiye case

The performance indicators are summarized in Table 13 for Türkiye case as pairwise comparison with effect group and official CCI. The fuzzy DEMATEL model limits the question set with five questions for Türkiye case. Therefore, the number of alternative CCIs constructed with the questions in cause group corresponds to 1452 while it corresponds to 13,116 alternative CCIs for the effect group.

**Table 13:** The performance comparison of models in literature with cause group for Türkiye case

Cause Group				
	The Number of Alternative CCIs	The % Change in The Number of Alternative CCIs	The Adjusted R^2 (Lasso Regression)	The % Change in Adjusted R^2 (Lasso Regression)
**Effect Group**	13,116	−88.93	0.46	79.97
**Official CCI**	480	202.50	0.63	33.04

Given that the performance of effect group, the cause group will provide 88.93% decreases in the number of alternative CCIs while provide 79.97% increases in explaining household consumption. Our model shows better performances in both performance indicators when compared to the effect group. The official CCI has the advantage about the low number of alternative CCIs. However, our model provides an increase in the prediction power of household consumption expenditures.

## 6 Conclusion

Since four of the CSs' twelve questions are thought to be indicative of consumer confidence, it is customary to use them in the CCI calculation while finding the perfect set of questions is still up for debate, Jonsson and Linden [9], Claveria et al. [27,28], as well as Lolic et al. [10]. The EC changed two of the questions that were used to determine the ranking even in 2019. This might be viewed as a continuing search for the optimal collection that can adequately describe household consumption. The studies that have been published in the literature have used various combinations of the questions and evaluated how well they predicted household consumption [9,10]. However, considering all possible combinations of questions has proven to be challenging since it takes too much time and processing resources.

This paper's central claim is that, as we'll see at the conclusion, some of the 12 CS questions are "causes" of changes in household consumption, while others are "effects" of those changes. This statement suggests that the questions in the "causes" category, rather than the "effects" category, are the most appropriate for characterizing shifts in household consumption. It will therefore be much easier to analyze fewer alternative confidence indexes if cause-and-effect relationships between the questions are found. With this purpose in the mind, we used a very reliable and robust methodology which is named as “Fuzzy DEMATEL”. This approach was applied both EU and Türkiye data sets to show its reliability and robustness.

The findings indicate that the questions in the cause group of EU case and the cause group of Türkiye case are completely different. The findings of EU case indicate that the questions measuring the assessments of consumers have a greater influence on household consumption when compared to the questions on expectations. Additionally, the household consumption is more impacted by micro-oriented inquiries than by macro-oriented ones. These two conclusions show differences for Türkiye case. The findings of Türkiye case indicate that the questions measuring the expectations of consumers have a greater influence on household consumption when compared to the questions on assessments. Additionally, macro-oriented inquiries have more impact on household consumption than micro-oriented ones.

The difference between the nature of cause group in EU case and Türkiye case support the idea of constructing country specific CCIs [27,28]. It also emphasizes the existing different mechanisms underlying household consumption and confidence amongst nations [9]. While the EU constructed by high income countries, Türkiye is upper-middle income country. Therefore, the economic diversity between the EU and Türkiye also affect the expectations and assessments of consumers about the general and household economy. Rapca and Chmielewska [48] summarized the results of researches on developed and developing countries as while the economically stable countries focused on assessments of the economic situation in the preceding period, the economically unstable countries focused on the expectations of respondents. The cause group questions content of the EU and Türkiye case are in line with this inference.

For EU case, the household consumption prediction performance of cause group, effect group, the question set of European CCI and the question set proposed in Lolic et al. [10] and the question set proposed in Jonsson and Linden [9] are compared with using lasso regression. The explanation power of cause group is better than the European CCI and the question set proposed in Jonsson and Linden [9]. The question set proposed in Lolic et al. [10] and the effect group show better performance than cause group in explaining household consumption. However, our proposed model has the advantage of being able to work with limited input data according to these models.

For Türkiye case, the household consumption prediction performance of cause group is compared with the effect group and the question set of official CCI. The cause group shows better prediction performance than effect group and official CCI. Therefore, it can be concluded that findings about the prediction performance of cause group of Türkiye case is in line with the result of EU case.

Since others select their best set of questions by experimenting different combinations with considerable number of computations, this work apparently proposes a shortcut of doing the same based on a logical argument. We evaluate the performance of our models according to two performance indicators which are “the number of possible alternative CCIs” and “the explanation power of household consumption”. For EU case, our model shows significant decreases in the number of possible alternative CCIs. Consequently, it also minimizes the demands on processing capacity and time during the analysis phase. Besides these advantages of our proposed model, it also shows good performances in explaining household consumption. For Türkiye case, our model increase the number of possible alternative CCIs compared the official CCI. However, it shows better prediction performance than official CCI. These factors make it reasonable to examine the cause-and-effect relationship between CS questions and household consumption when determining the ideal set of CS questions.

To the best of our knowledge, this study represents the first attempt to divide the set of CS questions into "Causes" and "Effects". This is also one of the few studies that examine the economic variables' component parts using the fuzzy DEMATEL method. By picturing these cause-and-effect relationships, we expect significant amount of computational saving to change focus from all combinations of questions to just combinations of causes (questions seen as the causes). The alternative confidence indicators can be constructed with different combination of the questions in cause group. The findings of our proposed models, which are constructed with using EU and Türkiye data, indicate the reliability and robustness of our approach.

Since, the mechanisms underlying household consumption and confidence may vary amongst nations [9], the cause group of EU case in our study may not be generalized to all EU member states and candidate states. This is the one of the limitations of our analysis. The difference between the cause group questions of EU case and Türkiye case also indicated this limitation. The EU constructed by high income countries. However the economic, demographic, and cultural diversities are existed among EU member states. From the total value of all goods and services produced in the EU, Germany has the largest share. On the other hand, according to the debt ratios, Greece, Italy, France, Spain, and Belgium have the highest debt rates [49]. This divergence would lead to differences between the expectations and assessments of consumers in these countries about the general and household economy. However, the results of the lasso regressions for EU and Türkiye cases indicated that the household consumption expenditures prediction power of the cause group is better than the official CCIs and other proposed question sets in the literature. Therefore, the fuzzy DEMATEL analysis can be conducted for each EU countries as a future study. Our approach would provide practical guidance for authorities to construct country specific CCIs.

The study's other limitation is that its computations only take linear relationships into account. The alternative consumer confidence indexes proposed by Claveria et al. [27,28] come into the question that the nonlinear relationships can be existed between CS questions. The genetic programming, which is one of the machine learning techniques, was used in the studies of Claveria et al. [27,28]. The neural networks can also learn and represent non-linear and complex connections between the input variables and target variables [50]. Therefore, the direct relationship between household consumption expenditures and CS questions can be considered with neural networks in future studies.

## CRediT authorship contribution statement

**Özge Var:** Data curation, Methodology, Investigation, Conceptualization, Formal Analysis, Writing – original draft, Writing – review & editing. **Alptekin Durmuşoğlu:** Methodology, Writing – original draft, Writing – review & editing, Supervision. **Türkay Dereli:** Supervision, Writing – review & editing.

## Data availability statement

The datasets were derived from sources in the public domain. The complete statistical dataset is available in the [EUROSTAT, https://ec.europa.eu/eurostat/web/main/data/database].

## Use of AI and AI-assisted technologies

AI technologies were not employed when creating this manuscript. The content was derived from the authors' interpretations of existing literature and informed discussions based on their expertise.

## Ethics statement

This article contains no studies performed by authors with human participants or animals. The publicly available and free-of-use data sets are used in the analysis.

## Funding statement

This research did not receive any specific grant from funding agencies in the public, commercial, or not-for-profit sectors.

## Declaration of competing interest

The authors declare that they have no known competing financial interests or personal relationships that could have appeared to influence the work reported in this paper.

## Figures and Tables

**Table 5:** The fuzzy pair wise comparison matrix (linguistic terms) for EU case

	C1	C2	C3	C4	C5	C6	C7	C8	C9	C10	C11	C12
**C1**	0	High Influence	High Influence	Very High Influence	Very High Influence	Very High Influence	Very High Influence	High Influence	Low Influence	High Influence	Low Influence	Low Influence
**C2**	Very Low Influence	0	Low Influence	High Influence	High Influence	High Influence	High Influence	Low Influence	Very Low Influence	Low Influence	Very Low Influence	Very Low Influence
**C3**	Very Low Influence	Low Influence	0	High Influence	High Influence	High Influence	High Influence	Low Influence	Very Low Influence	Low Influence	Very Low Influence	Very Low Influence
**C4**	No Influence	Very Low Influence	Very Low Influence	0	Low Influence	High Influence	Low Influence	Very Low Influence	No Influence	Very Low Influence	No Influence	No Influence
**C5**	No Influence	Very Low Influence	Very Low Influence	Low Influence	0	High Influence	Low Influence	Very Low Influence	No Influence	Very Low Influence	No Influence	No Influence
**C6**	No Influence	Very Low Influence	Very Low Influence	Very Low Influence	Very Low Influence	0	Very Low Influence	Very Low Influence	No Influence	Very Low Influence	No Influence	No Influence
**C7**	No Influence	Very Low Influence	Very Low Influence	Low Influence	Low Influence	High Influence	0	Very Low Influence	No Influence	Very Low Influence	No Influence	No Influence
**C8**	Very Low Influence	Low Influence	Low Influence	High Influence	High Influence	High Influence	High Influence	0	Very Low Influence	Low Influence	Very Low Influence	Very Low Influence
**C9**	Low Influence	High Influence	High Influence	Very High Influence	Very High Influence	Very High Influence	Very High Influence	High Influence	0	High Influence	Low Influence	Low Influence
**C10**	Very Low Influence	Low Influence	Low Influence	High Influence	High Influence	High Influence	High Influence	Low Influence	Very Low Influence	0	Very Low Influence	Very Low Influence
**C11**	Low Influence	High Influence	High Influence	Very High Influence	Very High Influence	Very High Influence	Very High Influence	High Influence	Low Influence	High Influence	0	Low Influence
**C12**	Low Influence	High Influence	High Influence	Very High Influence	Very High Influence	Very High Influence	Very High Influence	High Influence	Low Influence	High Influence	Low Influence	0

**Table 6:** Prominence, relation and weights of the criteria for EU case

	D_i_			R_j_			D_i_ + R_j_			D_i_-R_j_			(${\tilde{D}}_{i}^{\mathrm{def}}$ + ${\tilde{R}}_{j}^{\mathrm{def}}$)	(${\tilde{D}}_{i}^{\mathrm{def}}$ -${\tilde{R}}_{j}^{\mathrm{def}}$)	w_i_	W_i_
	l	m	h	l	m	h	l	m	h	l	m	h				
**C1**	1.2467	1.7682	2.8585	0.1304	0.4668	1.4413	1.3771	2.2350	4.2999	1.1163	1.3014	1.4172	2.5368	1.2841	2.8432	0.0868
**C2**	0.5217	1.1171	2.2533	0.5236	1.1180	2.2540	1.0454	2.2350	4.5073	−0.0019	−0.0009	−0.0008	2.5057	−0.0011	2.5057	0.0765
**C3**	0.5217	1.1171	2.2533	0.5236	1.1180	2.2540	1.0454	2.2350	4.5073	−0.0019	−0.0009	−0.0008	2.5057	−0.0011	2.5057	0.0765
**C4**	0.1667	0.5006	1.4891	1.1564	1.6928	2.7717	1.3231	2.1934	4.2609	−0.9898	−1.1921	−1.2826	2.4927	−1.1642	2.7511	0.0840
**C5**	0.1667	0.5006	1.4891	1.1564	1.6928	2.7717	1.3231	2.1934	4.2609	−0.9898	−1.1921	−1.2826	2.4927	−1.1642	2.7511	0.0840
**C6**	0.0000	0.3505	1.2817	1.4882	1.9762	3.0997	1.4882	2.3267	4.3814	−1.4882	−1.6257	−1.8180	2.6308	−1.6394	3.0998	0.0947
**C7**	0.1667	0.5006	1.4891	1.1564	1.6928	2.7717	1.3231	2.1934	4.2609	−0.9898	−1.1921	−1.2826	2.4927	−1.1642	2.7511	0.0840
**C8**	0.5217	1.1171	2.2533	0.5236	1.1180	2.2540	1.0454	2.2350	4.5073	−0.0019	−0.0009	−0.0008	2.5057	−0.0011	2.5057	0.0765
**C9**	1.2467	1.7682	2.8585	0.1304	0.4668	1.4413	1.3771	2.2350	4.2999	1.1163	1.3014	1.4172	2.5368	1.2841	2.8432	0.0868
**C10**	0.5217	1.1171	2.2533	0.5236	1.1180	2.2540	1.0454	2.2350	4.5073	−0.0019	−0.0009	−0.0008	2.5057	−0.0011	2.5057	0.0765
**C11**	1.2467	1.7682	2.8585	0.1304	0.4668	1.4413	1.3771	2.2350	4.2999	1.1163	1.3014	1.4172	2.5368	1.2841	2.8432	0.0868
**C12**	1.2467	1.7682	2.8585	0.1304	0.4668	1.4413	1.3771	2.2350	4.2999	1.1163	1.3014	1.4172	2.5368	1.2841	2.8432	0.0868

**Table 9:** The summary of lasso regression analyses

	The set of independent variables	R^2	Adjusted-R^2	F-statistic (prob)	Number of Selected Regressor	Selected Regressors Coefficient (prob)						
(a) EU case												
Cause Group	Q1, Q9, Q11,Q12	0.76	0.75	83.99 (0.000000)	2	Q1	Q11	_	_	_	_	_
						7380.994 (0.0029)	11814 (0.0000)	_	_	_	_	_
Effect Group	Q2, Q3, Q4, Q5, Q6, Q7, Q8, Q10	0.86	0.84	42.31 (0.000000)	7	Q2	Q3	Q4	Q5	Q6	Q8	Q10
						28934.37 (0.0000)	−4944.640 (0.0000)	−5210.529 (0.0003)	103.3951 (0.8997)	8164.268 (0.0000)	7717.107 (0.0026)	−9805.977 (0.0000)
European CCI	Q1, Q2, Q4, Q9	0.64	0.64	96.78 (0.000000)	1	Q1	_	_	_	_	_	_
						16989 (0.0000)	_	_	_	_	_	_
Lolic et al. [10]	Q1, Q3, Q4, Q6, Q8, Q9, Q10, Q11, Q12	0.89	0.88	100.64 (0.000000)	4	Q6	Q8	Q10	Q11	_	_	_
						3097.650 (0.0000)	831.7344 (0.4540)	−7671.276 (0.0000)	14967 (0.0000)	_	_	_
Jonsson and Linden [9]	Q3, Q8, Q9	0.68	0.66	36.64 (0.000000)	3	Q3	Q8	Q9	_	_	_	_
						−1687.106 (0.0532)	5837.627 (0.0041)	21453.07 (0.0000)	_	_	_	_

	The set of independent variables	**R^2**	**Adjusted-R^2**	**F-statistic (prob)**	**Number of Selected Regressor**	**Selected Regressors Coefficient (prob)**	
(b) Türkiye case							
Cause Group	Q2, Q4, Q5, Q6, Q8	0.86	0.84	36.76 (0.000000)	2	Q5	Q8
						16126.36 (0.4301)	−23840.81 (0.2519)
Effect Group	Q1, Q3, Q7, Q9, Q10, Q11, Q12	0.54	0.46	7.07 (0.009333)	2	Q3	Q11
						−25086.62 (0.1297)	−23809.13 (0.1845)
Official CCI	Q1, Q2, Q4, Q9	0.66	0.63	24.69 (0.000256)	1	Q2	_
						74184.73 (0.0003)	_

**Table 11:** Prominence, relation and weights of the criteria for Türkiye case

	D_i_			R_j_			D_i_ + R_j_			D_i_-R_j_			$\left({\tilde{D}}_{i}^{\mathrm{def}}+{\tilde{R}}_{j}^{\mathrm{def}}\right)$	$\left({\tilde{D}}_{i}^{\mathrm{def}}-\;{\tilde{R}}_{j}^{\mathrm{def}}\right)$	w_i_	W_i_
	l	m	h	l	m	h	l	m	h	l	m	h				
**C1**	0.2727	0.7000	2.0058	0.8928	1.4000	2.9408	1.1655	2.1000	4.9466	−0.6200	−0.7000	−0.9349	2.5780	−0.7387	2.6818	0.0817
**C2**	0.7622	1.3000	2.9073	0.2365	0.8000	2.1365	0.9987	2.1000	5.0438	0.5258	0.5000	0.7707	2.5606	0.5741	2.6242	0.0800
**C3**	0.2727	0.7000	2.0058	0.8928	1.4000	2.9408	1.1655	2.1000	4.9466	−0.6200	−0.7000	−0.9349	2.5780	−0.7387	2.6818	0.0817
**C4**	0.7622	1.3000	2.9073	0.2365	0.8000	2.1365	0.9987	2.1000	5.0438	0.5258	0.5000	0.7707	2.5606	0.5741	2.6242	0.0800
**C5**	1.4185	1.9000	3.3513	0.0370	0.2000	1.2351	1.4556	2.1000	4.5864	1.3815	1.7000	2.1162	2.5605	1.7244	3.0870	0.0941
**C6**	0.7622	1.3000	2.9073	0.2365	0.8000	2.1365	0.9987	2.1000	5.0438	0.5258	0.5000	0.7707	2.5606	0.5741	2.6242	0.0800
**C7**	0.2727	0.7000	2.0058	0.8928	1.4000	2.9408	1.1655	2.1000	4.9466	−0.6200	−0.7000	−0.9349	2.5780	−0.7387	2.6818	0.0817
**C8**	1.4185	1.9000	3.3513	0.0370	0.2000	1.2351	1.4556	2.1000	4.5864	1.3815	1.7000	2.1162	2.5605	1.7244	3.0870	0.0941
**C9**	0.2727	0.7000	2.0058	0.8928	1.4000	2.9408	1.1655	2.1000	4.9466	−0.6200	−0.7000	−0.9349	2.5780	−0.7387	2.6818	0.0817
**C10**	0.2727	0.7000	2.0058	0.8928	1.4000	2.9408	1.1655	2.1000	4.9466	−0.6200	−0.7000	−0.9349	2.5780	−0.7387	2.6818	0.0817
**C11**	0.2727	0.7000	2.0058	0.8928	1.4000	2.9408	1.1655	2.1000	4.9466	−0.6200	−0.7000	−0.9349	2.5780	−0.7387	2.6818	0.0817
**C12**	0.2727	0.7000	2.0058	0.8928	1.4000	2.9408	1.1655	2.1000	4.9466	−0.6200	−0.7000	−0.9349	2.5780	−0.7387	2.6818	0.0817
